# Characterizing the protein–protein interaction between MDM2 and 14-3-3σ; proof of concept for small molecule stabilization

**DOI:** 10.1016/j.jbc.2024.105651

**Published:** 2024-01-16

**Authors:** Jake A. Ward, Beatriz Romartinez-Alonso, Danielle F. Kay, Jeddidiah Bellamy-Carter, Bethany Thurairajah, Jaswir Basran, Hanna Kwon, Aneika C. Leney, Salvador Macip, Pietro Roversi, Frederick W. Muskett, Richard G. Doveston

**Affiliations:** 1 Leicester Institute for Structural and Chemical Biology, University of Leicester, Leicester, UK; 2 Mechanisms of Cancer and Aging Laboratory, Department of Molecular and Cell Biology, University of Leicester, Leicester, UK; 3 Department of Molecular and Cell Biology, University of Leicester, Leicester, UK; 4 School of Biosciences, University of Birmingham, Edgbaston, Birmingham, UK; 5 School of Chemistry, University of Leicester, Leicester, UK; 6 FoodLab, Faculty of Health Sciences, Universitat Oberta de Catalunya, Barcelona, Spain; 7 Josep Carreras Leukaemia Research Institute, Ctra de Can Ruti, Camí de les Escoles, s/n, Badalona, Barcelona, Spain; 8 Institute of Agricultural Biology and Biotechnology, C.N.R., Unit of Milan, Milano, Italy

**Keywords:** protein protein interaction, 14-3-3 proteins, MDM2, p53, molecular glue

## Abstract

Mouse Double Minute 2 (MDM2) is a key negative regulator of the tumor suppressor protein p53. MDM2 overexpression occurs in many types of cancer and results in the suppression of WT p53. The 14-3-3 family of adaptor proteins are known to bind MDM2 and the 14-3-3σ isoform controls MDM2 cellular localization and stability to inhibit its activity. Therefore, small molecule stabilization of the 14-3-3σ/MDM2 protein–protein interaction (PPI) is a potential therapeutic strategy for the treatment of cancer. Here, we provide a detailed biophysical and structural characterization of the phosphorylation-dependent interaction between 14-3-3σ and peptides that mimic the 14-3-3 binding motifs within MDM2. The data show that di-phosphorylation of MDM2 at S166 and S186 is essential for high affinity 14-3-3 binding and that the binary complex formed involves one MDM2 di-phosphorylated peptide bound to a dimer of 14-3-3σ. However, the two phosphorylation sites do not simultaneously interact so as to bridge the 14-3-3 dimer in a ‘multivalent’ fashion. Instead, the two phosphorylated MDM2 motifs ‘rock’ between the two binding grooves of the dimer, which is unusual in the context of 14-3-3 proteins. In addition, we show that the 14-3-3σ–MDM2 interaction is amenable to small molecule stabilization. The natural product fusicoccin A forms a ternary complex with a 14-3-3σ dimer and an MDM2 di-phosphorylated peptide resulting in the stabilization of the 14-3-3σ/MDM2 PPI. This work serves as a proof-of-concept of the drugability of the 14-3-3/MDM2 PPI and paves the way toward the development of more selective and efficacious small molecule stabilizers.

The transcription factor p53 plays a critical role in cell cycle regulation and tumor suppression (1). p53 inactivation *via* direct mutation or disruption of its regulatory network is a hallmark of many cancers (2, 3, 4). Therefore, p53 reactivation is a potentially powerful strategy for the development of antineoplastic drugs (5). In particular, the regulatory machinery that controls p53 homeostasis presents a number of targets for pharmaceutical intervention (5, 6). One such target has been the protein–protein interaction (PPI) between p53 and its negative regulator MDM2 (Mouse Double Minute 2; MDM2 is used herein to refer to the human protein) (7). MDM2 binds to the N-terminal transactivation domain of p53 to impede p53 interaction with DNA (8). In addition, MDM2 contains an E3 ubiquitin ligase domain that facilitates p53 ubiquitination and degradation *via* the proteasome (9, 10). Direct inhibition of the p53/MDM2 PPI using small molecules is an effective approach for restoring p53 activity in malignant cells, but this has not yet yielded any clinically approved anti-cancer therapies (5, 11). Targeting peripheral nodes of the p53 and/or MDM2 regulatory networks might provide a more tractable alternative or complimentary therapeutic strategy for p53 reactivation.

14-3-3 proteins represent one such node because they regulate MDM2 and p53 homeostasis *via* direct PPIs (6). 14-3-3 is a family of seven dimeric protein isoforms (β, γ, ε, ζ, η, σ, and τ) that integrates and controls multiple signaling pathways (12). 14-3-3 proteins modulate the enzymatic activity, subcellular localization, or interaction profile of over 1200 partner proteins *via* direct PPIs (13, 14). 14-3-3 proteins typically recognize consensus phosphorylated motifs within disordered regions of partner proteins. Three types of consensus phosphoserine (pS) or phosphothreonine (pT) peptide motifs are recognized by 14-3-3 proteins: mode I [RSX(pS/T)XP], mode II [RX(Y/F)X(PS/T)XP], or mode III [(pS/T)X-COOH] (15). These peptide motifs bind to an amphipathic groove that is characteristic of 14-3-3 proteins, whereby the phosphate group interacts with a conserved basic binding pocket formed by residues K49, R56, R129, and R127 (14-3-3σ numbering) (12). Although there is a high degree of sequence homology within the 14-3-3 family, each isoform exhibits its own affinity for the same binding partner (16, 17, 18), shows its own dimerization behavior (19, 20), and exerts its own physiological response (6, 12).

For example, four 14-3-3 isoforms directly interact with the p53 C terminus to positively regulate it in a manner dependent on Chk1/2 kinase activity (21). 14-3-3γ, ε, and ζ increase p53 transcriptional activity by promoting p53 tetramerization and DNA binding, whereas 14-3-3σ achieves the same by increasing the half-life of p53 in the cell (21). Promoting 14-3-3/p53 PPIs using small molecules is, therefore, a potential therapeutic strategy for p53 reactivation and approaches for achieving this have been explored (22, 23).

14-3-3 proteins also regulate MDM2. 14-3-3σ binds to MDM2 *via* a phosphorylation-dependent interaction in addition to what is presumed to be a phosphorylation-independent interaction with the C-terminal RING domain of MDM2 (24). This interaction negatively regulates MDM2 activity by promoting MDM2 auto-ubiquitination and degradation and by sequestering MDM2 from the nucleus into the cytoplasm (Fig. 1, panel *A*) (24). This leads to stabilization of p53 levels and enhances p53 transcriptional activity (24). Thus, the 14-3-3σ/MDM2 PPI is also a potential therapeutic target for drug molecules that would stabilize the interaction and promote tumor suppression.

**Figure 1:**
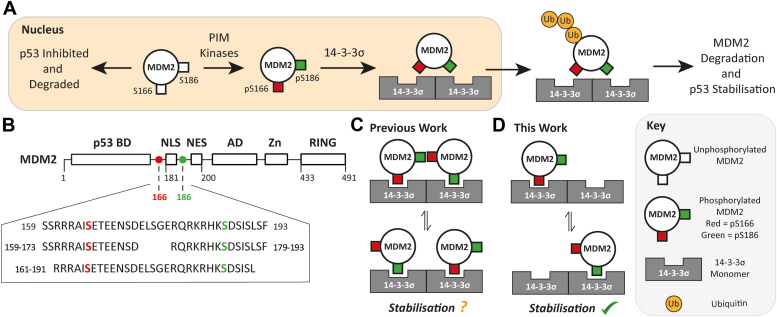
**The role of 14-3-3σ in MDM2 regulation.***A*, 14-3-3σ interacts with phosphorylated MDM2 to promote its degradation and p53 stabilization. *B*, MDM2 structure and 14-3-3 binding motifs. *C* and *D*, proposed mechanistic models for the 14-3-3σ/MDM2 PPI. MDM2, Mouse Double Minute 2; PPI, protein–protein interaction.

The specific MDM2 residues and kinases that facilitate MDM2 binding to 14-3-3σ have not been identified through cellular investigations. The phosphorylated motifs that bind to 14-3-3 family proteins with highest probability based on analysis of published examples using the *14-3-3Pred* webserver (25) are sequences that incorporate pS166 and pS186 (Fig. 1*B*). These do not represent typical recognition motifs, but the prediction is consistent with S166 and S186 phosphorylation by PIM kinases being a necessary condition for MDM2 binding to 14-3-3β, γ, ε, ζ, η, and τ isoforms (26). The physiological consequences of the interaction of MDM2 with these specific 14-3-3 isoforms have not been elucidated. However, in MDM2, the 14-3-3 binding motifs flank a nuclear localization sequence (Fig. 1*B*). It is therefore likely that all 14-3-3 isoforms influence MDM2 cellular localization, even though 14-3-3 isoform–specific profiles (other than for 14-3-3σ) have not yet been characterized.

In a recent study by Srdanović *et al.*, fluorescence polarization (FP) experiments provided the first *in vitro* evidence that all 14-3-3 isoforms, including 14-3-3σ, bind to peptides mimicking the pS166 and pS186 MDM2 motifs (17). The MDM2 peptides consistently bound to 14-3-3η with the highest affinity, and this isoform was used in further characterization studies involving isothermal titration calorimetry (ITC) and surface plasmon resonance (17). 14-3-3σ showed the lowest affinity for the MDM2 pS166 and pS186 peptides. It is not uncommon for the σ isoform to form weaker interactions with phosphorylated peptides relative to the other isoforms (16). This might further point to the importance of the aforementioned phosphorylation-independent secondary interaction involving the MDM2 RING domain. FP showed that the pS166 MDM2 peptide bound all 14-3-3 isoforms with a higher affinity than the pS186 peptide (17). A crystal structure of 14-3-3σ in complex with the lower affinity pS186 MDM2 motif showed a peptide bound in each of the two binding grooves available in the 14-3-3 dimer with similarities to a mode I binding pose (but lacking the +1 proline residue) (17). Significantly higher affinities for all 14-3-3 isoforms were observed using an MDM2 peptide with phosphorylation at both S166 and S186 sites (17). The higher affinity of di-phosphorylated peptides can be a result of the peptide simultaneously occupying both available binding grooves and bridging the 14-3-3 dimer in a ‘multivalent’ fashion (*i.e.* 2:1 14-3-3 monomer to peptide stoichiometry). However, ITC data indicated that one peptide occupied each available 14-3-3 binding groove (*i.e.* 1:1 stoichiometry, Fig. 1*C*) (17). Thus, it was proposed that high affinity binding of MDM2 for 14-3-3 proteins is driven by cooperativity between the two phosphorylation sites, a model described as ‘statistical rebinding’ (Fig. 1*C*) (17).

‘Multivalency’, where two or more phosphorylated motifs are required for high affinity binding, is integral to a number of 14-3-3 PPIs such as those with CFTR (27), LRRK2 (28), tyrosine hydroxylase (29), IRSp53 (30), PKCε (31), Nth1 (32), caspase-2 (33), MDMX (17), and MDM2 (17). Such systems are usually heteroditopic and feature a ‘gatekeeper’ or ‘anchor’ phosphorylation site that binds 14-3-3 first, followed by the second site (29, 31, 34). The affinity of such interactions is dependent on the individual affinities of the two sites and the effective molarity of the interacting partner (34). Such models assume that the two binding sites are identical, that is, the 14-3-3 dimer does not exhibit any cooperative behavior. It should be noted that the interactome profile of monomeric 14-3-3 protein differs from WT dimeric species indicating the physiological significance of the 14-3-3 dimer in partner protein recognition (35).

In this manuscript, we report data indicating that the interaction between 14-3-3σ and the pS166/pS186 MDM2 peptide is neither multivalent nor driven by ‘statistical rebinding’. A comprehensive biophysical and structural characterization using a combination of FP, ITC, native mass spectrometry (MS), protein X-ray crystallography, and NMR reveals that, in contrast to the previous report, the pS166/pS186 MDM2 peptide binds to 14-3-3σ *via* 2:1 stoichiometry, which is expected for a ‘multivalent’ interaction. However, the data also indicate that both phosphorylation sites do not simultaneously occupy a binding groove, and the peptide does not bridge the 14-3-3 dimer. The findings point to an unusual ‘rocking’ binding mechanism involving both phosphorylation sites (Fig. 1*D*). We also present data demonstrating that this interaction is amenable to small molecule stabilization and therefore a viable drug target.

## Results and discussion

Synthetic phospho-peptides were used to mimic the previously described 14-3-3 recognition motifs within the MDM2 sequence (17, 26). MDM2 phosphorylated at S166 or S186 was mimicked by a mono-phosphorylated 15 amino acid peptide spanning residues 159-173 and 179-193, respectively: MDM2_159-173_^pS166^ and MDM2_179-193_^pS186^ (Fig. 1*B*). A di-phosphorylated 31 amino acid peptide (MDM2_161-191_^pS166/pS186^) was used to mimic MDM2 with phosphorylation at both sites (Fig. 1*B*). The peptides were all amidated at the C terminus and either acetylated or fluorescently labeled at the N terminus.

### MDM2 di-phosphorylation is required for high affinity binding to 14-3-3σ

**Figure 2:**
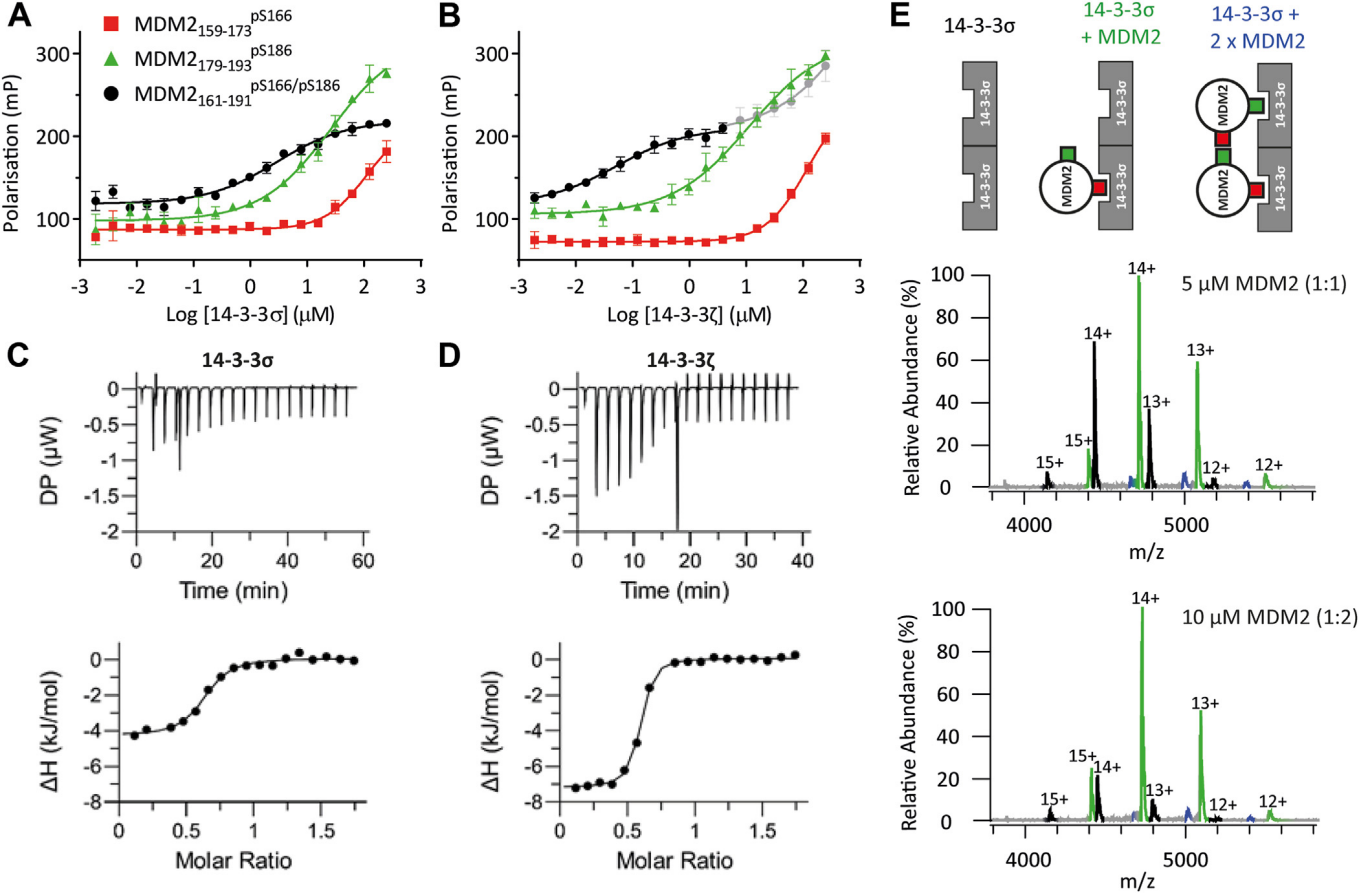
**Biophysical investigation of the 14-3-3σ/MDM2 PPI.***A* and *B*, FP data for MDM2 peptide binding to 14-3-3σ (*A*) and 14-3-3ζ (*B*, the second binding event is shown in *gray*). 14-3-3 protein was titrated to 10 nM FITC-labeled peptide in buffer containing 25 mM Hepes pH 7.5, 100 mM NaCl, 10 mM MgCl_2_, 0.1% v/v Tween20, 0.1 mg/ml BSA, and 1% v/v DMSO. Error bars represent SD for n = 3 replicates. *C* and *D*, ITC data for MDM2 peptide binding to 14-3-3σ (*C*) and 14-3-3ζ (*D*). Acetylated MDM2_161-191_^pS166/pS186^ peptide (1.0 mM) was titrated to 14-3-3 protein (0.1 mM) at 25 °C in buffer containing 25 mM Hepes pH 7.5, 100 mM NaCl, 10 mM MgCl_2_, and 1% v/v DMSO. The TFA content of the peptide was determined by ^19^F-NMR in order to accurately calculate peptide concentration (see Fig. S5 and Supporting information for details). *E*, native mass spectrometry data confirming 2:1 stoichiometry of 14-3-3σ monomer to MDM2 peptide. All protein concentrations are given as 14-3-3 monomer concentrations. FP, fluorescence polarization; ITC, isothermal titration calorimetry; MDM2, Mouse Double Minute 2; PPI, protein–protein interaction.

FP experiments were used to determine the relative binding affinities of the peptides for 14-3-3σ (Figs. 2, panel *A* and S1). 14-3-3σ was titrated to a fixed concentration of the fluorescently labeled peptides resulting in an increase in polarization as the 14-3-3σ–MDM2 binary complex formed. The MDM2_159-173_^pS166^ peptide bound weakly to 14-3-3σ and saturation was not observed. As a result, an effective K_d_ could only be estimated to be > 115 μM. The MDM2_179-193_^pS186^ peptide showed stronger binding to 14-3-3σ, but again an accurate curve could not be fitted. An approximate K_d_ was estimated to be 26.2 ± 17.9 μM. In agreement with the previous study (17), the di-phosphorylated MDM2_161-191_^pS166/pS186^ peptide exhibited significantly higher affinity binding to 14-3-3σ (effective K_d_ = 2.9 ± 1.0 μM).

The maximum polarization level observed for the MDM2_161-191_^pS166/pS186^ peptide was lower than that for the MDM2_179-193_^pS186^ peptide (Figs. 2*A* and S1). This suggests that the N-terminal fluorophore of the MDM2_161-191_^pS166/pS186^ peptide has greater rotational flexibility when bound to 14-3-3σ than the shorter MDM2_179-193_^pS186^ peptide. This might suggest that the C-terminal pS186 site indeed has a higher affinity for 14-3-3 than the pS166 site as indicated by the initial FP data.

In the previous study, it was shown that other 14-3-3 isoforms, for example, 14-3-3ζ, bound with higher affinity than 14-3-3σ to phosphorylated MDM2 peptide motifs (17). To corroborate this, 14-3-3ζ was titrated to fixed concentrations of the three peptides (Figs. 2*B* and S1). As with 14-3-3σ, the MDM2_159-173_^pS166^ peptide bound weakly to 14-3-3ζ, and no effective K_d_ value could be obtained. The MDM2_179-193_^pS186^ peptide bound to 14-3-3ζ with approximately a 2-fold higher affinity than the σ isoform (effective 14-3-3ζ K_d_ = 11.36 ± 5.4 μM vs 14-3-3σ K_d_ = 26.2 ± 17.9 μM). The MDM2_161-191_^pS166/pS186^ peptide again bound with significantly higher affinity, and in line with the previous study, a biphasic curve was observed (17). Biphasic binding curves for 14-3-3 PPIs have only been reported for MDM2 and MDMX peptides through FP studies (17). The observation is difficult to rationalize because FP only reports on the global average of all binding events that take place with a significant change in mass, hence ‘effective’ K_d_. Thus, the biphasic curve cannot be related to peptide-protein binding events. It could be related to nonspecific reduction of fluorophore rotational freedom or higher order complex formation at high protein concentrations, but further investigation is required. Nevertheless, assuming the monophasic binding curve observed for 14-3-3σ reflects the overall weaker affinity of this isoform for the MDM2_161-191_^pS166/pS186^ peptide, the effective K_d_ for 14-3-3ζ in the first phase was 56-fold lower than that for 14-3-3σ (51.9 ± 28.3 nM vs. 2.9 ± 1.0 μM). The effective K_d_ for the second phase could not be obtained because the curve did not reach saturation.

Further FP experiments were conducted to confirm that di-phosphorylation was a requirement for high affinity of the MDM2_161-191_^pS166/pS186^ peptide to 14-3-3σ. 14-3-3σ was titrated to 35 amino acid peptides with single phosphorylation sites at pS166 and pS186: MDM2_159-193_^pS166^ and MDM2_159-193_^pS186^. The longer MDM2_159-193_^pS166^ peptide showed a small increase in the maximum polarization value obtained upon 14-3-3σ titration compared to the 15 amino acid MDM2_159-173_^pS166^ peptide (Fig. S2). As before, saturation was not observed and an effective K_d_ value could not be obtained. The longer MDM2_159-193_^pS186^ peptide bound to 14-3-3σ with much weaker affinity than the 15 amino acid MDM2_179-193_^pS186^ peptide. Saturation was not observed and an effective K_d_ value could only be estimated to be >124 μM (Fig. S2). Therefore, di-phosphorylation and not peptide length accounted for the higher affinity of the MDM2_161-191_^pS166/pS186^ peptide. A noteworthy observation was that the maximum polarization level observed for the longer MDM2_159-193_^pS186^ peptide was comparable to that observed for the di-phosphorylated MDM2_161-191_^pS166/pS186^ peptide. In contrast, the shorter MDM2_179-193_^pS186^ peptide exhibited a much higher maximum polarization value (*vida supra*). These data further point to increased rotational flexibility of the N-terminal fluorophore when pS186 but not pS166 is anchored in the 14-3-3σ phosphate-binding pocket.

To confirm that the MDM2_161-191_^pS166/pS186^ peptide was engaging in a specific interaction with the 14-3-3σ binding groove, competition FP experiments were performed. The N-terminally acetylated analog of the MDM2_161-191_^pS166/pS186^ peptide was titrated to a fixed concentration of fluorescently labeled MDM2_161-191_^pS166/pS186^ and 14-3-3σ. As the concentration of the unlabeled peptide increased, polarization decreased because the fluorescent tracer peptide was competed out of the binding site (Fig. S3). The unlabeled MDM2_161-191_^pS166/pS186^ peptide had an IC_50_ of 3.2 ± 0.5 μM. A peptide mimicking the mode III 14-3-3 binding motif of the ERα transcription factor was also investigated. The 14-3-3/ERα PPI has been fully characterized, and this peptide motif is known to engage with the 14-3-3σ binding groove (36). This peptide also competed for binding to 14-3-3σ with the fluorescently labeled MDM2_161-191_^pS166/pS186^ tracer peptide with an IC_50_ of 1.5 ± 0.2 μM (Fig. S3). Thus, it can be concluded that the MDM2_161-191_^pS166/pS186^ peptide binds to the 14-3-3σ binding groove in a manner that depends on 14-3-3 recognition of MDM2 with phosphorylation at S166 and S186.

ITC provided orthogonal conformation of the K_d_ values obtained and further insight into the thermodynamics and stoichiometry of the MDM2 peptide interactions with 14-3-3σ and ζ. In these experiments, N-acetylated MDM2 peptides were titrated to a fixed concentration of 14-3-3 protein. Binding of the mono-phosphorylated MDM2_159-173_^pS166^ and MDM2_179-193_^pS186^ peptides could not be detected (Fig. S4). However, ITC could detect MDM2_161-191_^pS166/pS186^ peptide binding to 14-3-3σ with a K_d_ of 1.5 ± 0.4 μM (Figs. 2*C*, S5, and S6) and to 14-3-3ζ with ∼4-fold higher affinity (K_d_ = 0.38 ± 0.053 μM, Fig. 2*D*), values comparable with the FP data (Table 1).§ In these experiments, biphasic binding was not observed for 14-3-3ζ. This could be due to thedifferent concentration regimes used in ITC *versus* FP or could be due to the absence of the fluorophore in the ITC experiments. The higher affinity of the peptide for 14-3-3ζ was a result of a more negative enthalpy contribution relative to 14-3-3σ. Overall however, the interaction of the MDM2_161-191_^pS166/pS186^ peptide with both 14-3-3 isoforms was entropically driven (Table 1). This is interesting because the interactions of di-phosphorylated peptides that span the 14-3-3 dimer and simultaneously engage both binding grooves are typically enthalpy-dominated (37). The data seem to suggest that the MDM2 peptide binds to the 14-3-3 dimer in more than one way, binding induces a conformational change, and/or that MDM2 peptide binding displaces previously ordered water molecules in the binding groove.

**Table 1:** K_d_ values and thermodynamic parameters for MDM2_161-191_^pS166/pS186^ peptide binding to 14-3-3σ and 14-3-3ζ

Parameter	14-3-3σ	14-3-3ζ
K_d_ (FP, μM)	2.9 ± 1.0	(1) 0.052 ± 0.028 (2) -
K_d_ (ITC, μM)	1.5 ± 0.4	0.38 ± 0.053
ΔG (kJmol^−1^)	−33.3	−36.7
N	0.60 ± 0.013	0.55 ± 0.003
ΔH (kJmol^−1^)	−4.4 ± 0.2	−7.2 ± 0.1
−TΔS (kJmol^−1^)	−28.9	−29.4

### One MDM2 di-phosphorylated peptide binds to a 14-3-3 dimer

The ITC experiments showed that the stoichiometry (N) for the interactions of the MDM2_161-191_^pS166/pS186^ peptide with 14-3-3σ and 14-3-3ζ to be 0.60 and 0.55, respectively (Table 1, Figs. 2, panels *C* and *D*,and S6).§ This is a strong indication that the 14-3-3 dimer interacts with a single di-phosphorylated MDM2_161-191_^pS166/pS186^ peptide, that is, 2:1 ratio of 14-3-3 monomer to peptide. These data disagreed with that from the previous study which reported 1:1 binding stoichiometry (17). To confirm the ITC data, native MS experiments were conducted.

Native MS allows for the analysis of proteins and protein complexes without denaturing protein secondary structure or disrupting noncovalent interactions (38). Stoichiometry and binding equilibria can be determined from the unique mass of the complexes involved (39). MS analysis revealed that 14-3-3σ was almost exclusively dimeric at a concentration of 5 μM (Fig. S7). Upon the addition of an equimolar concentration of the MDM2_161-191_^pS166/pS186^ peptide, a binary complex was observed (Fig. 2*E*). The binary complex consisted of the 14-3-3σ dimer and a single MDM2_161-191_^pS166/pS186^ peptide. When a higher concentration of the MDM2_161-191_^pS166/pS186^ peptide was added, the population of the binary complex increased, and the population of apo-14-3-3σ decreased (Fig. 2*E*). There was scant evidence of a complex consisting of a 14-3-3σ dimer and two MDM2_161-191_^pS166/pS186^ peptides and that which was observed is most likely a result of nonspecific binding (40). Therefore, the native MS data confirm that the interaction of 14-3-3σ with the MDM2_161-191_^pS166/pS186^ peptide has a stoichiometry of 2:1. This contrasts to native MS analysis of other 14-3-3σ PPIs, such as ERα, which involve 1:1 binding of phospho-peptide to 14-3-3 monomer (39). In these cases, a distribution of apo 14-3-3σ monomer, 2:1 and 1:1 stoichiometry is observed, representing an equilibrium that shifts toward 1:1 as the peptide concentration is increased or a stabilizer is added. This is not observed in the case of the MDM2_161-191_^pS166/pS186^ peptide.

### Structural analysis of the 14-3-3σ/MDM2 PPI

**Figure 3:**
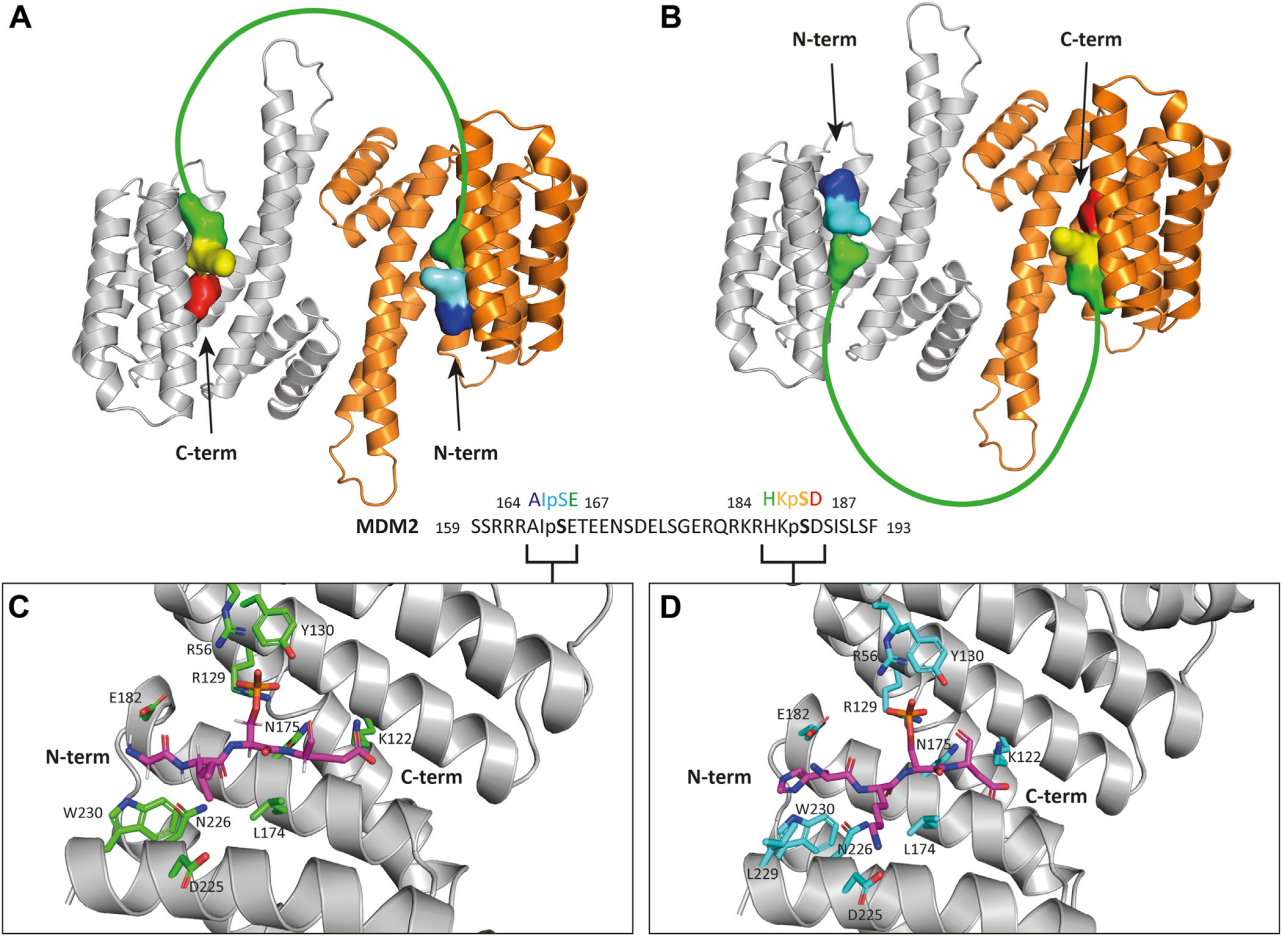
**Crystallographic analysis of the 14-3-3 interaction with the MDM2**_**161-191**_^**pS166/pS186**^**peptide (PDB:****8P0D****).***A* and *B*, the 14-3-3σ dimer in the crystal, in cartoon representation, with one monomer shown in *gray* and one monomer in *orange*. The complex sits in the same site in two different orientations (*A* and *B*) causing the spatial overlap of the two ordered regions of the MDM2 peptide in the crystal. The latter are represented as surfaces in panels A and B. The 164-167 stretch of the MDM2 peptide is colored *blue* to *green* from N terminus to C terminus; the 184-187 stretch of the MDM2 peptide is colored *green* to *red* from N terminus to C terminus. The disordered part of the MDM2 peptide, 168-183, is represented as a *green* line. *C* and *D*, the two overlapping ordered stretches of the MDM2 peptide bound in the 14-3-3σ binding groove. N atoms are shown in *blue*, O atoms in *red*. Peptide: in sticks with C atoms in magenta. 14-3-3: backbone in *gray* cartoon representation; side chains that interact with the peptide in sticks representation with C atoms in *green* or *cyan*. *C*, the MDM2 164-167 stretch. *D*, the MDM2 184-187 stretch. MDM2, Mouse Double Minute 2.

A crystal structure of the MDM2_161-191_^pS166/pS186^ peptide in complex with a 14-3-3σ ΔC construct (lacking the final 17 residues at the C-terminus) was determined to a resolution of 1.3 Å (Tables S1 and S2). The complex of the 14-3-3σ dimer shows density for a single peptide occupying both 14-3-3σ binding grooves (Fig. 3, panels A and B). In the crystal, the asymmetric complex sits at the same site (around a crystal twofold axis) in two different orientations, so that the ordered parts of the N-terminal (residues ^164^AIpSE^167^) and C-terminal (^184^HKpSD^187^) portions of the peptide overlap (static disorder, Fig. 3, panels *A* and *B*). The peptide binds in the expected orientation with its C-terminal end directed toward the N terminus of 14-3-3σ. Both pS166 and pS186 phosphorylation sites are engaged with the 14-3-3σ binding groove, interacting with the residues that form the phosphate-binding pocket: R56, R129, and Y130. The −2 and +1 residues relative to either pS are also well ordered in the crystal (Fig. 3, panel *C* and *D*).

MDM2 peptide main chain atoms interact with the side chains of 14-3-3σ N175 and N226 and, together with the pS side chain interactions, define the pose of the peptide backbone around the phosphorylation site in a MDM2 sequence-independent manner. Polar interactions are observed between main chain O and N atoms of MDM2 D157 and E167 and the side chain of 14-3-3σ N175 and between main chain O and N atoms of MDM2 I165 and K185 and the side chain of 14-3-3σ N226.

Further to these MDM2 peptide backbone interactions, there are some MDM2 peptide sequence-dependent side chain interactions. In the ^164^AIpSE^167^ part of the peptide, a hydrophobic contact is observed between the side chains of MDM2 I165 and 14-3-3σ L229 while MDM2 E167 forms a salt bridge with 14-3-3σ K122. H-bonds are observed between the side chains of MDM2 H184 and 14-3-3σ Y181, E182, and W230 and between the side chains of MDM2 K185 and 14-3-3σ D225; MDM2 D187 forms a salt bridge with 14-3-3σ K122. The interactions of the ^184^HKpSD^187^ part of the peptide confirm the ones already observed in PDB ID 6YR6^17^ and more broadly both peptides bind in a similar way to other 14-3-3 client peptides (see Fig. S9). The structure was deposited with PDB ID 8P0D.

Protein NMR was used to further investigate the stoichiometry and dynamics of the 14-3-3σ/MDM2 PPI. Backbone chemical shift assignments (^15^N, ^13^C, ^1^H) have previously been reported for the 14-3-3σ ΔC construct (41) and the C-terminal domain of 14-3-3σ(42). This enabled ^1^H-^15^N transverse relaxation optimized spectroscopy (TROSY) spectra to be collected for ^15^N-enriched 14-3-3σ (full length), and the observed resonances were assigned to specific amino acid residues. Only a single resonance is observed for each amide proton/nitrogen pair in the spectrum indicating that 14-3-3σ is observed as a symmetric dimer in solution (although two distinct complexes could be distinguished by crystallography, the system is under fast exchange, and the two complexes would be equivalent in solution).

**Figure 4:**
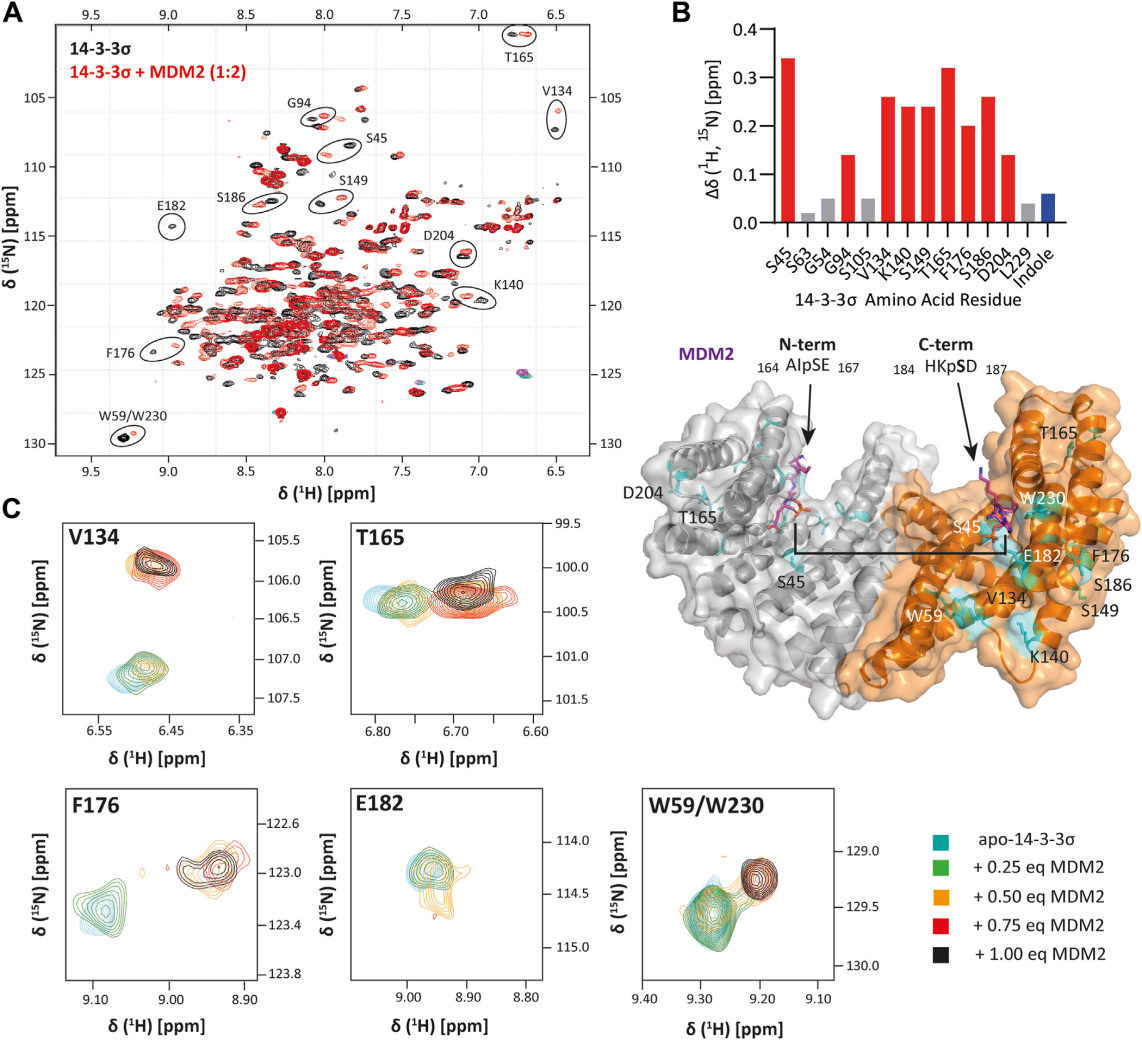
^**1**^**H-**^**15**^**N TROSY NMR analysis of the 14-3-3σ interaction with the MDM2**_**161-191**_^**pS166/pS186**^**peptide.***A*, superimposed ^1^H-^15^N TROSY spectra of ^15^N-labeled apo-14-3-3σ (100 μM, *black*) and ^15^N-labeled 14-3-3σ (100 μM) in complex with the MDM2_161-191_^pS166/pS186^ peptide (200 μM) (*red*). All protein concentrations are given as 14-3-3 monomer concentrations. *B*, *top*: Plot showing the notable combined ^1^H and ^15^N chemical shift perturbations in the spectrum of ^15^N-labeled 14-3-3σ (100 μM) in complex with the MDM2_161-191_^pS166/pS186^ peptide (200 μM). Residues displaying significant CSPs are shown in *red*, the CSP relating to the indole resonance is shown in *blue*. For reference, the CSP for three residues that did not show notable changes are shown in *gray*. *B*, *bottom*: CSPs (*cyan*) mapped onto the protein X-ray crystal structure of 14-3-3σ ΔC dimer in complex with the MDM2_161-191_^pS166/pS186^ peptide (*purple*). The disordered part of the MDM2 peptide, 168-183 is represented as a *black* line. *C*, selected ^1^H-^15^N TROSY resonances for 14-3-3σ (100 μM) in the presence of increasing concentrations of the MDM2_161-191_^pS166/pS186^ peptide (see key). MDM2, Mouse Double Minute 2.

^1^H-^15^N TROSY spectra were collected on 14-3-3σ in complex with the MDM2_161-191_^pS166/pS186^ peptide and compared to the spectrum of free 14-3-3σ. In the presence of 2 M equivalents of peptide relative to 14-3-3σ monomer, distinct chemical shift perturbations (CSP) were observed (Fig. 4, panel *A* and *B*). There were no additional differences in the CSPs observed when 2.5 M equivalents of peptide relative to 14-3-3σ monomer were used (Fig. S10). This corroborates the ITC data that indicates binding saturation at 2 M equivalents of peptide. It provides further evidence for 2:1 14-3-3σ monomer-peptide binding stoichiometry because a greater molar excess of peptide would be expected to be required to reach saturation if two peptides were simultaneously engaging a 14-3-3σ dimer. The symmetry of the system was however retained upon peptide binding as shown by the single distinct set of resonances observed. This suggests that the MDM2_161-191_^pS166/pS186^ peptide engages with both monomers *via* a fast exchange mechanism.

CSPs were mapped onto the crystal structure of 14-3-3σ bound to the MDM2_161-191_^pS166/pS186^ peptide (Fig. 4*B* and Table S3). CSPs were observed for four amino acids within, or proximal to, the 14-3-3σ binding groove: S45, V134, F176, and S186. The most significant perturbation was observed for S45 which lies at the N-terminal end of the binding groove and the crystal structure indicates that S45 forms long hydrogen bonds with the carboxylate side chains MDM2 E167 and E187. V134 lies at the C-terminal entrance to the binding groove and faces the main 14-3-3 dimer interface. Thus, it is possible the CSP results from the MDM2 peptide spanning the length of the binding groove and perhaps distortion of the dimer upon binding. F176 lies next to N175 which is observed to form a polar contact with the MDM2 peptide backbone in the crystal structure. S186 also lies at the C-terminal entrance to the binding groove and is observed to point away from the peptide in the crystal structure, perhaps indicating this residue side chain rotates upon peptide binding.

There were notable CSPs for two resonances within the indole region of the spectra. 14-3-3σ contains two tryptophan residues bearing indole side chains (W59 and W230). It was not possible to assign the resonances to specific residues based on the reported backbone assignment. However, both W59 and W230 are located in helices that form the 14-3-3σ binding groove (Fig. 4*B*). The indole side chain of W230 protrudes into the binding groove and in the crystal structure is observed to form a hydrogen bond with the side chain of the MDM2 peptide H184 residue, which is consistent with the previously reported structure with a mono-phosphorylated MDM2^pS186^ peptide (17). The side chain of W59 is proximal to that of V134 for which the corresponding resonance also undergoes CSP—both form part of the 14-3-3σ dimer interface.

In addition to CSPs, the resonance corresponding to E182, which is also located in the binding groove, broadened such that it was not visible in the spectrum of the 14-3-3σ–MDM2 complex. This is also indicative of an interaction between the amino acid residue and the peptide. It is consistent with the crystallography data that shows E182 forms a polar contact with the MDM2 peptide which is again consistent with the previous report (17).

CSPs were not discernible for the key residues that form the phosphate-binding pocket, as observed from the crystal structure (R56, R129, Y130). R129 and Y130 were not assigned in the original backbone assignment of 14-3-3σ(41), and so signals for these residues were not identified in our spectra. A small CSP upon phosphopeptide binding for R56 has been reported previously (42), but because it lies in a densely populated region of the spectrum, it could not be measured from our data.

CSPs were also observed for residues distal to the binding groove: G94, K140, S149, T165, D204 (Fig. 4, panels *A* and *B* and Table S3). CSPs for G94 and S149 have been previously reported for 14-3-3σ in complex with a peptide motif mimicking the Tau protein (42). These CSPs could reflect conformational changes to 14-3-3σ structure upon peptide binding. T165 and D204 are seen to form hydrogen bond in the crystal structure of the binary complex. Together, the NMR data confirmed that the MDM2_161-191_^pS166/pS186^ peptide was binding in the 14-3-3σ groove.

To probe the dynamics of the 14-3-3σ–MDM2 interaction, a titration experiment was conducted. ^1^H-^15^N TROSY spectra of 14-3-3σ were collected in the presence of increasing concentrations of the MDM2_161-191_^pS166/pS186^ peptide. Intensities of resonances corresponding to apo-14-3-3σ decreased as the concentration of peptide was increased. Conversely, resonances corresponding to the 14-3-3σ–MDM2 complex increased as the concentration of peptide was increased, with the exception of that for E182 which broadened such that it could no longer be observed. This was most notable for residues V134, T165, F176, E182, and one of the tryptophan indoles (W59 or W230, Fig. 4*C*). Saturation of 14-3-3σ was observed when 1 M equivalent of peptide was introduced relative to the 14-3-3σ monomer (*i.e.* 100 μM 14-3-3σ and 100 μM MDM2_161-191_^pS166/pS186^ peptide). This provides further evidence for 2:1 monomer-peptide binding stoichiometry, again because a greater excess of peptide would be expected to be required to reach saturation if two peptides were simultaneously engaging a 14-3-3σ dimer. For residues V134, T165, and either W59 or W230, two distinct resonances were observed when 0.5 M equivalents of peptide were introduced relative to the 14-3-3σ monomer (*i.e.* 100 μM 14-3-3σ and 50 μM MDM2_161-191_^pS166/pS186^ peptide, Fig. 4*C*). This is intriguing because it indicates that the two states observed in the ^1^H-^15^N TROSY spectra are in slow exchange on the NMR timescale. These two states may reflect a 14-3-3σ conformational change that is induced by MDM2 peptide binding. As only one set of NMR signals (bound and free) are observed for each residue during the titration, symmetry of the dimer is preserved throughout, indicating that the MDM2 peptide binding event itself is in fast exchange between the two available binding grooves present in the 14-3-3 dimer. This complex and dynamic binding mechanism could relate directly to the entropy-driven nature of the interaction as shown by ITC. Furthermore, the conformational change induced upon MDM2 peptide binding could be responsible for the biphasic FP curves as a result of a reduction in fluorophore rotational freedom.

### The 14-3-3σ/MDM2 PPI is amenable to small molecule stabilization

Stabilization of the 14-3-3σ/MDM2 PPI using ‘molecular glues’ (*i.e.* small molecules that enhance the affinity of a PPI by forming a ternary protein–protein–drug complex) could be an effective strategy for enhancing p53 tumor suppressor activity. To investigate the feasibility of this, the effect of fusicoccin A (FC-A) on the PPI was studied using FP, ITC, and native MS. FC-A is a fungal metabolite that predominantly stabilizes mode III 14-3-3 PPIs whereby the binary complex creates a ligand-binding pocket (43). However, FC-A has also been shown to stabilize peptides containing mode I/II type di-phosphorylated binding motifs such as that for CFTR (27).

**Figure 5:**
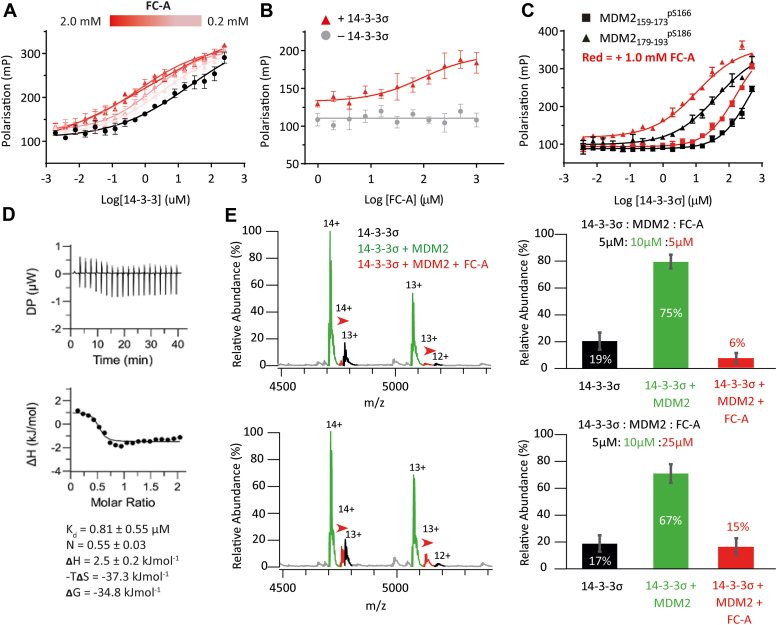
**Stabilization of the 14-3-3/MDM2 PPI by FC-A.***A*, FP data for MDM2_161-191_^pS166/pS186^ peptide binding to 14-3-3σ in the presence of FC-A. 14-3-3σ was titrated to 10 nM FITC-labeled peptide and increasing fixed concentrations of FC-A. *B*, FP dose-response data for FC-A. FC-A was titrated to fixed concentration of 14-3-3σ (1 μM) and FITC-labeled peptide (10 nM). *C*, FP data for MDM2_159-173_^pS166^ and MDM2_179-193_^pS186^ peptide binding to 14-3-3σ. 14-3-3σ was titrated to 10 nM FITC-labeled peptide and 1.0 mM FC-A. All FP experiments were conducted in buffer containing 25 mM Hepes pH 7.5, 100 mM NaCl, 10 mM MgCl_2_, 0.1% v/v Tween20, 0.1 mg/ml BSA, and 1% v/v DMSO. Error bars represent SD for n = 3 replicates. *D*, ITC data for MDM2_161-191_^pS166/pS186^ peptide binding to 14-3-3σ in the presence of FC-A. Acetylated MDM2_161-191_^pS166/pS186^ peptide (1.0 mM) was titrated to 14-3-3σ protein (0.1 mM) in the presence of FC-A (1.0 mM) at 25 °C in buffer containing 25 mM Hepes pH 7.5, 100 mM NaCl, 10 mM MgCl_2_, and 1% v/v DMSO. *E*, native mass spectrometry data showing the FC-A concentration-dependent formation of a ternary complex. *Red* arrows highlight peaks for the ternary complex. Error bars represent SD for n = 3 replicates. FP, fluorescence polarization; ITC, isothermal titration calorimetry; MDM2, Mouse Double Minute 2; PPI, protein–protein interaction.

FP experiments were conducted to establish if FC-A increased the affinity of the MDM2_161-191_^pS166/pS186^ peptide for 14-3-3σ. 14-3-3σ was titrated to a fixed concentration of the fluorescently labeled MDM2_161-191_^pS166/pS186^ peptide in the presence of increasing concentrations of FC-A (Figs. 5, panel *A* and S11). The effective K_d_ decreased in a FC-A concentration-dependent manner from 3.5 ± 2.1 μM in the control experiment to a minimum of 0.60 ± 0.10 μM in the presence of 1.0 mM FC-A. This represented a 5.8-fold stabilization of the 14-3-3σ/MDM2 PPI. Titration of FC-A to a fixed concentration of 14-3-3σ and the MDM2_161-191_^pS166/pS186^ peptide tracer also showed FC-A dose-dependent stabilization (EC_50_ = 67.1 ± 41.1 μM; Figs. 5*B* and S11).

To determine if FC-A had a preferential effect on either phosphorylation site, further 14-3-3σ titrations were carried out using the fluorescently labeled monophosphorylated MDM2_159-173_^pS166^ and MDM2_179-193_^pS186^ peptides (Figs. 5*C* and S11). FC-A had a small effect on the 14-3-3σ interaction with the weakly binding MDM2_159-173_^pS166^ peptide whereby the binding curve was shifted slightly to the left, and a higher maximum polarization value was obtained. As before, saturation was not reached and accurate fitting data were not obtained. FC-A did have a significant effect on the 14-3-3σ interaction with the MDM2_179-193_^pS186^ peptide. FC-A (1.0 mM) stabilized this interaction by 2.5-fold (K_d_ = 24.6 ± 12.0 μM → 10.8 ± 2.9 μM). The maximum polarization value obtained also increased slightly, likely as a result of conformational restraint of the peptide N terminus.

ITC was used to further investigate FC-A stabilization of the 14-3-3σ interaction with the MDM2_161-191_^pS166/pS186^ peptide. The N-terminal acetylated peptide was titrated to a fixed concentration of 14-3-3σ in the presence of FC-A (Fig. 5*D*). In alignment with the previous experiments, the stoichiometry of the interaction was 2:1, 14-3-3 monomer to MDM2 peptide. However, in stark contrast to the binary system, the interaction of the MDM2 peptide with 14-3-3σ in the presence of FC-A was an endothermic process entirely dominated by an entropic contribution which could again be explained by a significant hydrophobic effect or changes in protein structure (Fig. 5*D*). The K_d_ for the interaction in the presence of FC-A could not be accurately measured but did not appear to change significantly, perhaps reflecting the modest stabilizing effect: K_d_ = 0.81 ± 0.55 μM, compared to 1.5 ± 0.4 μM for the binary system. Nevertheless, this is a ∼2-fold stabilization that is consistent with the effect seen by FP. Binding was entirely driven by the entropic component which could be a result of a large hydrophobic effect or of residual structural disorder in the ternary complex.

To confirm that FC-A was engaged in a ternary complex with 14-3-3σ and the MDM2 peptide, a series of native MS experiments were conducted. In the presence of 2 M equivalents of MDM2 peptide, a distribution of 14-3-3σ dimer and 14-3-3σ/MDM2 binary complex was observed (Fig. 5*E*). Upon addition of FC-A, a ternary FC-A/MDM2/14-3-3σ complex was formed which comprised a 14-3-3 dimer, a single MDM2 di-phosphorylated peptide, and a single FC-A molecule. As the concentration of FC-A increased, the abundance of the ternary complex also increased to a modest degree (6 ± 3% → 15 ± 5%; Figs. 5*E* and S8). These data confirm that FC-A forms a ternary complex with 14-3-3σ and MDM2 *via* binding to a single site—the FP data indicate this is most likely the interface of the MDM2 pS186 motif with 14-3-3σ. Furthermore, the data indicate that FC-A modestly promotes complex formation in a concentration-dependent manner.

## Conclusion

Here, we report on a biophysical and structural characterization of the 14-3-3σ/MDM2 PPI which is an important node in MDM2 and p53 homeostasis and a potential target for therapeutic intervention using ‘molecular glues’.

FP data corroborated those reported in a previous study by Srdanović *et al.* (17), confirming that phosphorylation of both MDM2 S166 and S186 residues, which flank a nuclear localization sequence of MDM2, are essential for high affinity phosphopeptide binding to 14-3-3σ and 14-3-3ζ. Neither the S166 nor S186 sites are located within canonical 14-3-3 recognition motifs, highlighting the versatility of 14-3-3 molecular recognition. In this study, we observed the pS186 site to bind with slightly higher affinity than the pS166 site, which contrasts with the previous report (17). However, the affinities of both mono-phosphorylated peptides are very low, and it was not possible to determine accurate apparent K_d_ values. This suggests that binding of MDM2 to 14-3-3 does not involve a higher affinity ‘gatekeeper’ phosphorylation site and a secondary low affinity phosphorylation site.

ITC showed that a peptide mimicking the di-phosphorylated MDM2 motif bound *via* an entropy-driven process, again in agreement with the data reported by Srdanović *et al.* (17). This is unusual in the context of other 14-3-3 PPIs whereby binding of di-phosphorylated motifs tend to be enthalpically driven when a ‘multivalent’ binding mechanism is invoked (*i.e.* whereby the peptide spans the 14-3-3 dimer with simultaneous engagement of both phospho-sites) (37). Therefore, this finding strongly suggests that the MDM2-derived di-phosphorylated peptide is not engaged in ‘multivalent binding’. Instead, binding appears to be driven by a significant hydrophobic effect or a change in protein secondary structure upon peptide binding.

Our ITC and native mass spectrometry data show that a single MDM2 peptide binds to a 14-3-3σ dimer (2:1 stoichiometry). This contrasts with ITC data reported in the previous study that indicated two MDM2 peptides occupied the 14-3-3η dimer (*i.e.* 1:1 stoichiometry) (17). The contrast in results is most likely explained by a difference in behavior between the two 14-3-3 isoforms investigated in the two studies. 14-3-3σ (studied here) is reported to be almost exclusively homodimeric, while 14-3-3η has been reported to exist as a 7:3 mixture of dimeric to monomeric forms (19). The contrasting data sets are intriguing because they highlight 14-3-3 isoform-specific binding profiles which are poorly understood at the molecular level and warrant further investigation.

Novel protein crystallographic analysis of 14-3-3σ in complex with the MDM2 di-phosphorylated peptide confirmed that both the pS166 and pS186 can bind to the 14-3-3 binding groove. However, the data indicates that the peptide does not bridge the 14-3-3σ dimer with both phosphosites simultaneously bound to each monomeric unit. Furthermore, there is no apparent preference for binding of the pS166 site over the pS186 site. This contrasts with other reported di-phosphorylated binding motifs, including that of MDMX (17), which do have a higher affinity ‘gatekeeper’ residue, and bind in a multivalent fashion (see multivalency discussion above).

NMR was used to further elucidate the binding mechanism for the MDM2-derived di-phosphorylated peptide to 14-3-3. This revealed that, on the NMR timescale, the symmetry of the 14-3-3 dimer was retained upon peptide binding, thus further ruling out a multivalent binding mechanism which would have led to desymmetrization. Therefore, in solution, the peptide likely ‘rocks’ between each monomer groove *via* a fast exchange mechanism. This highlights the potential significance of cooperativity between 14-3-3 monomers in controlling certain PPIs, something that could be closely related to isoform-specific behaviors (*vide supra*). NMR also revealed that MDM2 peptide binding induces a conformational change in the structure of 14-3-3σ which is under a slow exchange mechanism. This is significant because it again highlights the dynamic nature of 14-3-3 proteins.

The physiological implications of this unusual binding mechanism could be to mask the nuclear localization sequence on MDM2 leading to cytoplasmic accumulation. The dependency of MDM2 di-phosphorylation for high affinity 14-3-3 binding, and a lack of a clear ‘gatekeeper’, may suggest an ‘all or nothing’ binding response. This supports previously reported cellular studies that show, while S186 is rapidly phosphorylated by Pim kinases, 14-3-3 only engages MDM2 when S166 also becomes phosphorylated over a longer timescale, presumably in response to an increase in cellular stress (26). However, it is not clear how this interaction promotes MDM2 auto-ubiquitination—this may relate to the aforementioned secondary interaction with the RING domain of MDM2.

Stabilization of the 14-3-3σ/MDM2 PPI could be an effective strategy for promoting/protecting p53 tumor suppressor activity. Stabilization of this oncosuppressive interaction may have several advantages over classical MDM2 inhibition. A key issue surrounding inhibition of the MDM2/p53 PPI is an increase in MDM2 protein levels, due to MDM2 being a transcriptional target of p53 (44). This increase in MDM2 creates a 'sink' for inhibitors, which can create a limit to the extent the p53 pathway can be activated. Based on the available biological evidence, stabilization of the 14-3-3σ/MDM2 may result in MDM2 degradation (24). Degradation of MDM2 has recently been shown to indeed dampen the negative feedback mechanism between MDM2 and p53 when compared with MDM2 inhibitors (45). Here, we use FP, ITC, and native MS to demonstrate proof-of-concept for small molecule stabilization of this PPI using FC-A. Although the stabilizing effect of FC-A is modest, the FP data indicate that a ligand-binding site is available at the interface of 14-3-3σ and the pS186 motif of MDM2. Thus, the data provide a valuable foundation for further structure-based design efforts to more efficacious and selective molecular glues for this important PPI.

## Experimental procedures

For full details on preparation and characterization of reagents, peptides, and proteins used in this study, please refer to the supporting information.

### Fluorescence polarization

Fluorescence polarization experiments were conducted at room temperature in buffer containing: 25 mM Hepes pH 7.5, 100 mM NaCl, 10 mM MgCl_2_, 0.1% v/v Tween20, 0.1 mg/ml BSA, and 1% v/v DMSO using Corning black, round-bottom, low-binding 384-well plates. For all experiments, a fixed concentration of 10 nM fluorescently labeled peptide (FITC) was used. All plates were incubated for 10 min and shaken for 10 s before polarization was measured using a Hidex Sense Microplate reader with an excitation wavelength of λex: 490/20 nm; and an emission wavelength of λem: 535/20 nm; mirror dichoric 560; flashes: 20; PMT voltage 750; Z-position: calculated from well. All data was analyzed using GraphPad Prism 7 and sigmoidal curves were fitted using the following equation: Y=Bottom + (Top-Bottom)/(1+10ˆ((Log app.Kd-X)∗HillSlope)), where Y = mP value; X = log 14-3-3 concentration; top and bottom = plateaus in mP.

### Isothermal titration calorimetry

ITC experiments were conducted using the Malvern MicroCal ITC200 instrument (monophosphorylated peptides) or Malvern PEAQ ITC instrument (di-phosphorylated peptide). The ITC conditions used are detailed below in Table S5. All experiments were conducted in buffer containing 25 mM Hepes pH 7.5, 100 mM NaCl, 10 mM MgCl_2_, and 1% v/v DMSO. Where two titrations were conducted, the data was merged using Concat32 software (Malvern Instruments Ltd) All data was analyzed using MicroCal PEQ-ITC Analysis Software (single site binding model) to obtain parameters (where applicable).

### Native MS

14-3-3 σ was buffer exchanged into 100 mM ammonium acetate pH 6.8 using a 30 kDa molecular weight cut-off Amicon Ultra centrifugal filter (Merck Millipore) and stored at −80 °C prior to use. The lyophilized MDM2_161-191_^pS166/pS186^ 31mer peptide (see Table S4) was diluted into 100 mM ammonium acetate pH 6.8. To form the 14-3-3–MDM2 complex, 14-3-3σ (5 μM) was mixed with 5 μM and 10 μM MDM2 31mer di-phosphorylated peptide. To form the stabilized 14-3-3–MDM2 complex, 14-3-3σ (5 μM) was incubated with both a 2-fold excess of the MDM2 31mer di-phosphorylated peptide (10 μM) and FC-A (both 5 μM and 25 μM) in 100 mM ammonium acetate pH 6.8 and directly infused into the mass spectrometer. A final concentration of 1.25% DMSO was used for all experiments.

All native MS experiments were performed on an Orbitrap Eclipse Tribrid mass spectrometer (Thermo Fisher Scientific, Bremen) coupled to a nanoelectrospray source that used gold-coated borosilicate glass capillaries, pulled in-house. Positive ionization mode was used throughout with the capillary voltage set to 1.2 kV. The source temperature was set at 275 °C, in-source dissociation at 25, S-lens RF at 100. High pressure mode was used and a mass range of 2000 to 8000 *m/z* used to monitor the binding equilibria. Mass spectra were acquired using a maximum ion injection time of 50 ms. The automatic gain control was set to 1 x 10^6^ and the ions detected in the Orbitrap with resolution set to 7500. All data was analyzed using XCalibur (*v*.4.1). All proteins and protein complexes were identified based on their theoretical mass.

### Protein X-ray crystallography

For crystallization, 10 mg/ml of 14-3-3σ ΔC17 was mixed with MDM2 peptide in a 1:1 M ratio in 25 mM Hepes pH 7.5, 100 mM NaCl, 10 mM MgCl_2_ and incubated overnight at 4 °C. Vapor diffusion crystallization 200 nl sitting drops were set up using a Mosquito crystallization robot (SPT Labtech), mixing the 14-3-3σ ΔC17/MDM2 peptide complex in mother liquor in the following volume ratios: 1:1 (drop 1) and 1:2 (drop 2), using a bespoke crystallography screen of pH and PEG concentrations with 1 M Hepes and 0.19 M CaCl_2_. All plates were stored refrigerated (4 °C). Crystals grew in drops 1 and 2 within a week at the following conditions: 1 M Hepes pH 7.4 to 7.6, 0.19 M CaCl_2_, 30 to 31% PEG 400, 5% glycerol. After 1 month, crystals were fished at 4 °C, flash cooled in liquid nitrogen, and exposed to X-rays.

For structure determination, the CCP4 (46) software package was used with PDB: 3IQJ (47) serving as a template of the 14-3-3σ structure for molecular replacement using PHASER (48). Further rounds of manual model building and refinement were performed using COOT (49) and REFMAC (50), respectively. Data collection and refinement statistics are shown in Tables S1 and S2. Crystallographic data has been deposited in the Protein Data Bank under accession code 8P0D.

### Nuclear magnetic resonance

All spectra were recorded from 100 μM samples of ^15^N-labeled HIS_6_-tagged full-length 14-3-3σ in buffer containing 25 mM Hepes, pH 7.5, 100 mM NaCl, 10 mM MgCl_2_, 3 mM sodium azide, and 5% (v/v) D_2_O in a 5 mm Shigemi tube (sample volume: 350 μl) using a Bruker 600 MHz AVIII spectrometer operating at 303K. The ^1^H^15^N TROSY spectra were recorded with acquisition times of 60 msec in the directly detected dimension and 40 msec on the indirect dimension, with 48 scans per increment (total acquisition time of ∼ 5 h). Spectra were collected for 14-3-3σ only and in the presence of different molar equivalence of MDM2 peptide (0.25, 0.5, 0.75, 1.0, 2.0, and 2.5 eq). Spectra were processed with Topspin 4.0.6 (Bruker Biospin) to produce the ^1^H^15^N TROSY spectra. ^1^H, ^15^N combined chemical shift changes upon binding were calculated as Δδ^1^H, ^15^N = |Δδ^1^H| + |0.2∗Δδ^15^N|, with Δ the difference between chemical shifts values δ between the apo-14-3-3σ resonances and those in the presence of 2.0 eq of MDM2 peptide.

## Data availability

The mass spectrometry data supporting this research is openly available from the 10.13039/501100000855University of Birmingham data archive at https://doi.org/10.25500/edata.bham.00000954. Crystallographic data has been deposited in the Protein Data Bank under accession code 8P0D. All other data are available on request from Richard G. Doveston, r.g.doveston@leicester.ac.uk.

## Supporting information

This article contains Supporting information (17, 27, 36, 46, 47, 48, 51).

## Conflict of interest

The authors declare that they have no conflicts of interest with the contents of this article.

## Footnotes

§ To accurately calculate peptide concentration the TFA content of the MDM2_161-191_^pS166/pS186^ peptide was determined by ^19^F-NMR (see Fig. S5 and Supporting information for details).
